# Microbial profile of the appendix niche in acute appendicitis: a novel sampling approach

**DOI:** 10.1002/2211-5463.70105

**Published:** 2025-08-22

**Authors:** Huimin Ma, Mingfei Wang, Yanhu Feng, Wanqi Zhang, Wenbo Wang, Yanfeng Xie, Guixiang Kong, Jie Feng, Pengfei Wang, Qi Wang, Xiaojun Huang

**Affiliations:** ^1^ The Second Hospital & Clinical Medical School Lanzhou University China; ^2^ Department of Gastroenterology The Second Hospital of Lanzhou University China; ^3^ Digestive Endoscopy Center The Second Hospital of Lanzhou University China; ^4^ Department of General Surgery The First Hospital of Lanzhou University China; ^5^ Cuiying Biomedical Research Center The Second Hospital of Lanzhou University China

**Keywords:** acute appendicitis, appendix niche, endoscopic retrograde appendicitis treatment, microbial profile, shotgun metagenomic sequencing

## Abstract

Relatively little is known about the microbial variations within the human appendix niche. To overcome this knowledge gap, we employed endoscopic retrograde appendicitis treatment (ERAT) technology to collect microbial samples from the appendix lumen, followed by shotgun metagenomic sequencing on participants with acute appendicitis without antibiotic treatment. Compared to the cecum and terminal ileum, the appendix had a higher abundance at the genus level of *Sphingobium*, *Leptotrichia* and *Oribacterium*, as well as a significant increase in species‐level abundance of oral bacteria, including *Streptococcus sanguinis*, *Streptococcus australis*, *Streptococcus* sp. A12, *Leptotrichia* sp. oral taxon 215, *Veillonella dispar*, *Veillonella infantium* and *Oribacterium sinus*. Pearson correlation analysis showed that bacterial species abundant in the appendix, such as *Acinetobacter johnsonii*, *Sphingobium yanoikuyae* and *Agrobacterium tumefaciens*, had negative correlations with the top five most abundant Gene Ontology (GO) categories (molecular function, biological process and cellular component). Conversely, species underrepresented in the appendix, including *Mogibacterium diversum*, *Streptococcus sanguinis*, *Megasphaera micronuciformis* and *Actinomyces graevenitzii*, had significant positive correlations with these GO categories. Our results show that ERAT technology can be used to improve sampling and microbiome profiling in the appendix. Furthermore, this in‐depth microbial characterization could inform clinicians during antibiotic prescription. However, further large sample size studies are required to validate these results.

## Graphical abstract

This study utilized a novel sampling method, ERAT (i.e. endoscopic retrograde appendicitis treatment)‐guided lumen aspiration, to obtain samples from the appendix, and shotgun metagenomic sequencing was performed for *in situ* characterization of the appendix microbiome in patients with acute appendicitis. Compared to the cecum and terminal ileum, the appendix showed a higher abundance of oral bacteria, and certain ‘beneficial bacteria’ were also detected.

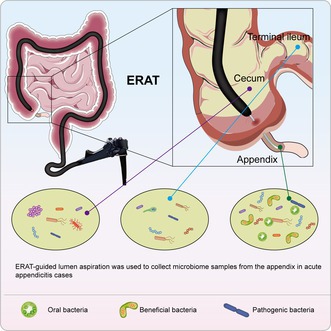

**Abbreviations**
BP: biological process
CC: cellular component
DNB: DNA nanoball
ERAT: endoscopic retrograde appendicitis treatment
GO: Gene Ontology
IBD: inflammatory bowel disease
MF: molecular function
PERMANOVA: permutational multivariate analysis of variance
SCFA: short‐chain fatty acid

Acute appendicitis represents the most prevalent abdominal surgical emergency globally, exhibiting an annual incidence rate ranging from 96.5 to 100 cases per 100 000 adults [1]. The clinical manifestation of acute appendicitis is typically characterized by migratory abdominal pain in the right lower quadrant [2]. The precise pathophysiology of appendicitis remains incompletely understood; however, luminal obstruction combined with bacterial translocation and subsequent infection are considered plausible underlying mechanisms [3, 4]. If the condition deteriorates, complications such as appendiceal perforation, abscess formation and peritonitis may occur [5]. The main treatments for appendicitis include appendectomy and antibiotic therapy [6]. Endoscopic retrograde appendicitis treatment (ERAT) has the capability to manage appendicitis without necessitating the removal of the appendix, making it especially suitable for simple appendicitis cases [7, 8].

The appendix is generally considered as a redundant organ, and the role of the appendix in immunity and microbial maintenance has long been a matter of debate. However, increasing evidence from research indicates that the appendix functions as an immune organ and concurrently serves as a microbial reservoir, thereby regulating gut microbiota [9, 10]. In rats with appendicitis, appendectomy can suppress the activity of Th17 cells, inhibit autophagy, modulate molecules associated with interferon activity and restrain endothelin‐mediated vascular remodeling [11]. In the T cell receptor‐α knockout rat model, which exhibits impaired regulation of local T and B cell proliferation, the number of IgA‐expressing B cells in the appendix‐associated gut‐associated lymphoid tissue (GALT) is significantly increased [12]. A study demonstrated that, in children with complicated appendicitis, enhanced Th17 responses in the appendix are linked to microbial dysbiosis, and that cytokines produced by CD4+ T cells, particularly interleukin‐17A, are markedly elevated [13]. Consequently, alterations in the microbiome residing within the appendix may contribute to the emergence and progression of appendicitis, as well as potentially to the related illnesses, such as colorectal cancer [14], diverticular disease [15], mental disorders [16], inflammatory bowel disease [17] and other disease [18, 19]. Currently, there is a scarcity of data regarding the microbial composition of the human appendix. It is concluded that the appendix harbors symbiotic bacteria that construct an efficient biofilm and potentially expedite the re‐establishment of colonic microbiota [20]. Jackson *et al*. [21] examined the elevated microbial composition in normal appendices from children and identified *Paenibacillaceae*, *Acidobacteriaceae GP4*, *Pseudonocardinae*, *Bergeyella* and *Rhizobium*. This analysis suggests the presence of distinct microbial populations within the appendix. Guinane *et al*. [22] identified the predominant bacterial phyla of the human appendix microbiome as Firmicutes, Proteobacteria, Bacteroidetes, Actinobacteria and Fusobacteria. Notably, there was a heightened prevalence of oral‐associated bacteria detected in inflamed appendices as opposed to their non‐inflamed counterparts [23, 24]. It is of particular significance to highlight that there exists a notable regional diversity in microbiota along the gastro‐intestinal tract [25]. Factors such as pH levels, regional oxygen concentration, nutrient availability and bile acids, which substantially influence microbial preferences, collectively contribute to the formation of region‐specific microbial communities [26]. In rats, the appendix may serve as a transitional zone involved in the modulation of microflora between the upper and lower intestines [27]. In the aye‐aye, the appendiceal microbiome has lower richness and diversity, greater evenness, and a unique taxonomic composition compared to the more homogeneous cecal and colonic communities [28]. However, there is a lack of comprehensive knowledge regarding the ecological differences of the appendix and its surrounding environment in humans. Furthermore, variations in research outcomes can be attributed to the sampling methodologies employed [29]. To date, no studies have documented the utilization of ERAT technology in conjunction with visual endoscopy for sampling within the appendix lumen. This sampling offers a robust technical foundation for comprehensive research into the appendix's micro‐environment.

To address the aforementioned unresolved issues, we have collected a total of 49 samples, comprising 20 patients, with patients contributing samples from appendix niche (including the appendix, the terminal ileum and the cecum). Utilizing the colonoscopy biopsy channels, we employed visual endoscope (Micro‐Tech; Nanjing Co., Ltd, Nanjing, China) to obtain samples from the appendix lumen and a modified spraying tube to collect the adjacent intestinal fluid, respectively. Subsequent shotgun metagenomic sequencing was conducted to investigate the microbiome within the appendix niche.

## Materials and methods

### Subjects and study design

This study aimed to investigate the microbiota characteristics within the human appendiceal niche by collecting samples from the appendix cavity, terminal ileum and cecum using ERAT techniques in patients diagnosed with acute appendicitis. Patients with severe complications such as appendix perforation, peritonitis and abscess, those who were intolerant to colonoscopy and ERAT surgery, and individuals who had received intravenous or oral antibiotics prior to surgery were excluded from this study.

**Fig. 1:**
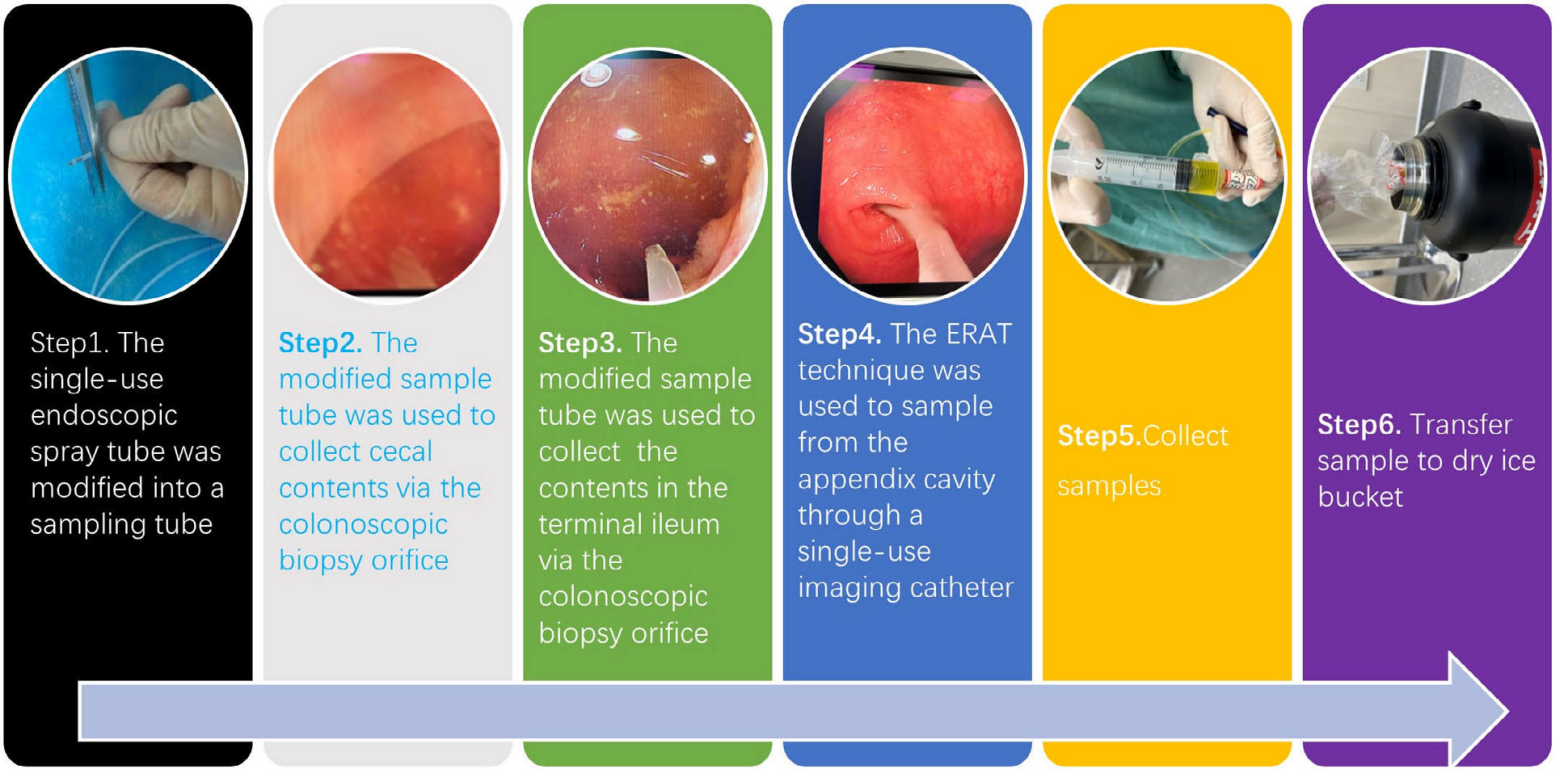
The sampling process of microbial samples collected from the appendix and the intestinal segment around the appendix by the ERAT technique. Step 1: the single‐use endoscopic spray tube was modified into a sampling tube. Step 2: the modified sample tube was used to sample from the cecum. Step 3: the modified sample tube was used to sample from the terminal ileum. Step 4: the ERAT technique was used to sample from the appendix cavity through a single‐use imaging catheter. Step 5: samples were collected. Step 6: samples were transferred to dry ice.

The study was conducted in accordance with the Declaration of Helsinki, and approved by the Ethics Committee of Lanzhou University Second Hospital (protocol code 2023A‐090; approval date 7 February 2023). Written informed consent was obtained from either the patient or their authorized representative. Questionnaires were designed to gather information on gender, age, height, weight, residential address, medication use, family history and dietary habits of the study participants (Table S1). All subjects underwent bowel preparation using a standardized intestinal cleansing protocol prior to the surgical procedure. The cleansing agent used was polyethylene glycol electrolyte powder (manufactured by Shenzhen Wanhe Pharmaceutical Co., Ltd, Shenzhen, China). One package of the compound was dissolved in 1 L of water and consumed in divided doses, and three packages were used in total until the stool became clear. All biological samples and clinical biochemical indicators were collected following standardized operating procedures at the Second Affiliated Hospital of Lanzhou University in Gansu Province (Fig. 1). Specifically, regarding the collection process of biological samples: they were promptly stored in a freezing chamber at −80 °C before being swiftly transported on dry ice to a designated laboratory.

### DNA preparation and metagenomics sequencing

The DNA extraction of intestinal contents samples was performed using the QIAamp Fast DNA Stool Mini Kit (Qiagen, Hilden, Germany). The procedure was conducted as follows: the sample was weighed and then 1.4 mL of Inhibit EX Buffer was added and mixed thoroughly using a vortex mixer until a homogeneous suspension was formed. The suspension was placed in a water bath at 95 °C for 5 min to heat the sample. This was followed by centrifugation at 4000×*g* for 1 min. Next, 200 μL of the supernatant was carefully pipetted into a new centrifuge tube and centrifuged again at 4000×*g* for 1 min. The supernatant was transfered to an Eppendorf (EP) tube containing 15 μL of proteinase K. Then, 200 μL of buffer AL was added to the EP tube containing the supernatant and proteinase K. This was followed by mixing gently and incubation in an incubator for 10 min at 70 °C. Next 200 μL of ethanol was added to the EP tube and mixed using a vortex mixer to ensure homogeneity. Then 600 μL of the sample liquid containing DNA was carefully loaded onto a QIAamp spin column and centrifuged at 8000×*g* for 1 min. The waste liquid was discarded from the collection tube. Sequentially 500 μL of buffer AW1 and 500 μL of buffer AW2 were added to the QIAamp spin column, centrifuging at 8000×*g* after each addition and discarding the filtrate from the collection tube. The QIAamp spin column was transfered to a 2‐mL collection tube and centrifuged for 3 min to remove any residual liquid. The QIAamp spin column was placed in a 1.5‐mL EP tube. Then, 200 μL of buffer AE was added the tube was allowed stand at room temperature for 1 min to fully saturate the DNA‐bound membrane. Centrifugation at 4000×*g* was performed to elute the DNA into the EP tube. Subsequently, DNase‐free RNase treatment was performed on extracts for assessing DNA quality using agarose gel electrophoresis and a Qubit 3.0 fluorimeter (Thermo Fisher Scientific, Waltham, MA, USA). After performing quality control tests for total DNA concentration and integrity, 500 ng of DNA was fragmented via ultrasound using a Covaris E220 (Covaris, Woburn, MA, USA), followed by size‐selection using magnet beads to obtain fragments ranging from 300 to 700 bp [30]. The selected DNA fragments were repaired and ligated with an indexed adaptor. The ligation products underwent PCR amplification and then 55 ng of purified PCR products were denatured at 95 °C and ligated by T4 DNA ligase (L603‐HC‐1500; Enzymatics, Beverly, MA, USA) at 37 °C to create a single‐strand circular DNA library, followed by hybridization with an exon probe and capture via streptavidin beads. The captured DNA was subjected to another round of PCR amplification and circularized to generate a single‐stranded circular library. This single‐stranded circular library was then amplified via rolling circle amplification to obtain DNA nanoballs (DNB) [31]. Subsequently, the DNBs were loaded onto a flowcell and sequenced utilizing a DNBSEQ platform (BGI‐Shenzhen, Shenzhen, China). Raw reads containing more than 95% low‐quality bases (quality ≤ 20) or over five ambiguous bases were excluded from further analysis, whereas the remaining reads underwent filtering procedures targeting host DNA elimination based on the reference human genome as previously described [32]. Metagenomics sequencing employing pair‐end libraries with read length of 150 bp was performed on the DNBSEQ‐T1 platform [31]. Quality control assessments were carried out along with adapter removal and host contamination filtration using fastp [33] and bowtie2 [34] (Table S2).

### Taxonomic and functional profiling acquisition

We used MetaPhlAn3 and HUMAnN 3.0 for taxonomic and functional profiling acquisition [35]. The taxonomic profiles were generated from high‐quality reads using Metaphlan 3.0, (‐input_type fastq‐ignore_viruses‐nproc6), as described on the official website (https://huttenhower.sph.harvard.edu/metaphlan3). MetaPhlAn relies on unique clade‐specific marker genes identified from approximately 17 000 reference genomes (approximately 13 500 bacterial and archaeal, approximately 3500 viral and 110 eukaryotic), allowing up to 25 000 reads‐per‐second (on one CPU) analysis speed. The normalization procedure was conducted in accordance with the methodology described in the literature [35]. For functional profiling, HUMAnN 3.0 (‐i input_clean_data ‐o output ‐‐threads 10 ‐‐memory‐use maximum ‐‐remove‐temp‐output) was employed to efficiently and accurately assess the abundance of microbial metabolic pathways and other molecular functions in the metagenomic sequencing data (clean data) based on the corresponding website (https://huttenhower.sph.harvard.edu/humann).

### Alpha‐ and beta‐diversity analysis

The alpha diversity [within‐sample diversity, R, version 4.0.3 vegan: diversity (data, index = ‘richness/shannon/Simpson’) (https://cran.r‐project.org/package=vegan)] was assessed by calculating the richness, Shannon index and Simpson index based on the taxonomic profiles using a *t*‐test and the Wilcoxon rank sum test [30]. *P* < 0.05 was considered significantly different.

The beta diversity [between‐sample diversity, R, version 4.0.3 ape: pcoa (‘bray_curtis distance’, correction = “none”, rn = NULL) (https://cran.r‐project.org/package=ape)] was determined using the Bray–Curtis distance with respect to the taxonomic profiles with the *t*‐test. To investigate the impact of group on the gut microbiome, a permutational multivariate analysis of variance [i.e. PERMANOVA; code: R, version 4.0.3: adonis (dist‐phe, permutations = 9999) (https://rdrr.io/rforge/vegan/man/adonis.html)] was conducted on the abundance profiles of gut microbial species, genus, pathway. *P* < 0.05 was considered significantly different.

### Statistical analysis

R, version 4.2.0 (R Core Team, 2018; R Foundation, Vienna, Austria) with Bioconductor 3.13 was employed for the analyses [36]. To characterize the clinical data, we summarized its statistical properties using measures such as mean and variance. For comparing alpha diversity between groups, we employed both the *t*‐test and the Wilcoxon rank sum test. To evaluate differences in bacterial abundances and their associations with functional pathways, we applied the Wilcoxon rank sum test and Spearman's correlation. For comparisons across three positions, we utilized the Kruskal–Wallis test. Multiple testing adjustments were performed using the Benjamini–Hochberg method (code: *P*. adjust (*P* value, method = “BH”)). *P* < 0.05 was considered significantly different.

## Results

In total, 20 participants diagnosed with acute appendicitis, who did not receive antibiotic treatment, were recruited for this study (Fig. 1). Clinical data of the participants were collected, revealing minimal significant differences across various statistical analyses. These data included age, sex, white blood cell count, neutrophil count, C‐reactive protein levels, clinical types of appendicitis (simple or complex), appendiceal orifice inflammation and Alvarado score (Table S1). Notable statistical variations were observed in the clinical classifications of appendicitis during pathway analysis. Additionally, disparities were identified in the bacterial genera present in the cecum when comparing different genders (Table S3).

### Characteristics and the comparison of the gut microbiota in the appendix and its adjacent intestinal sites

**Fig. 2:**
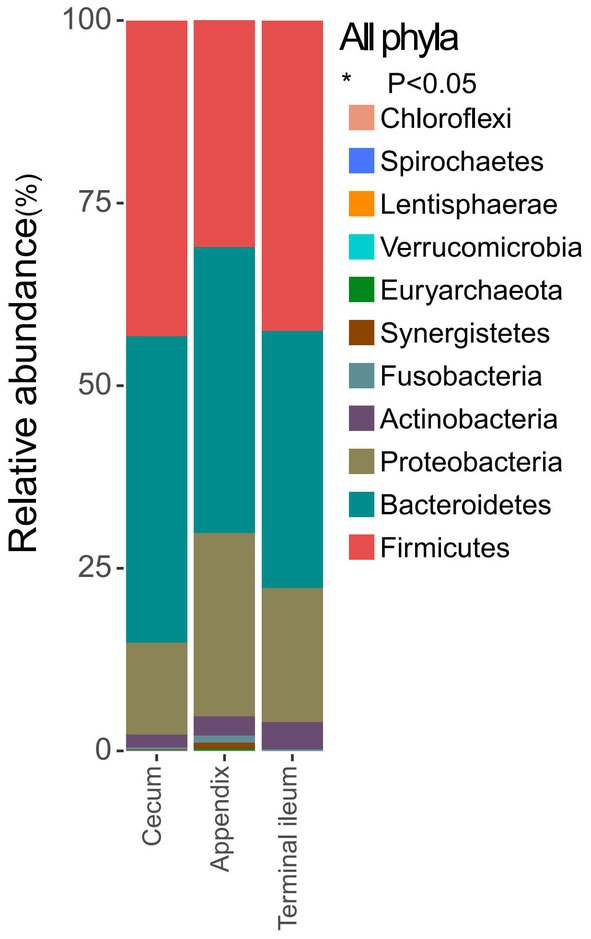
The top 10 abundant phyla in the appendix, the cecum and the terminal ileum (*P* < 0.05): Firmicutes, Bacteroidetes, Proteobacteria, Actinobacteria, Fusobacteria, Synergistetes, Euryarchaeota, Verrucomicrobia, Lentisphaerae and Spirochaetes.

In the appendix and its neighboring sites, such as the cecum and terminal ileum, the top 10 abundant phyla (Table S4) were Firmicutes, Bacteroidetes, Proteobacteria, Actinobacteria, Fusobacteria, Synergistetes, Euryarchaeota, Verrucomicrobia, Lentisphaerae and Spirochaetes (Fig. 2).

**Fig. 3:**
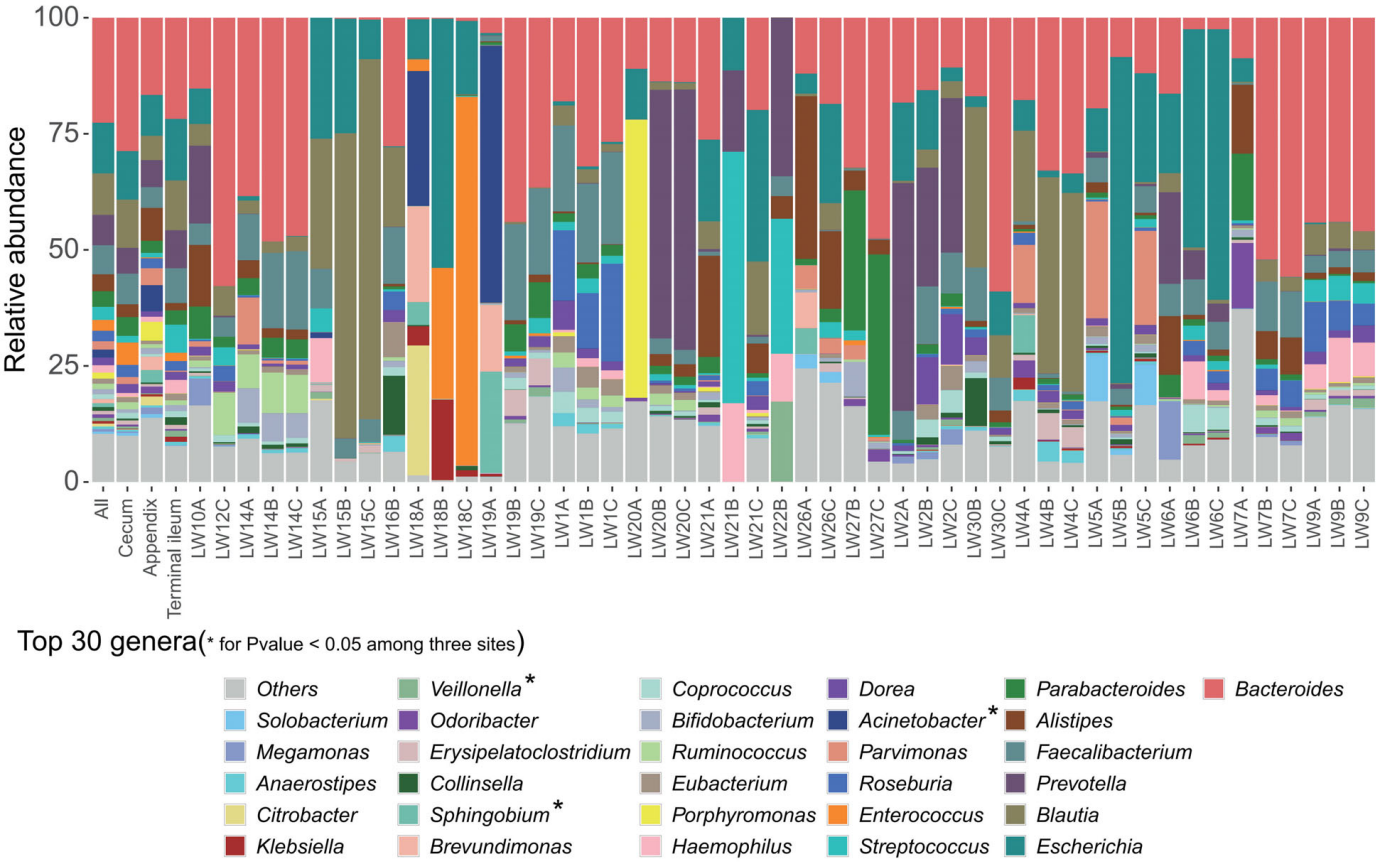
The top 30 genera across the appendix, the cecum and the terminal ileum. *Veillonella*, *Sphingobium* and *Acinetobacter* exhibited statistically significant differences (Kruskal–Wallis test, *P* < 0.05).

**Fig. 4:**
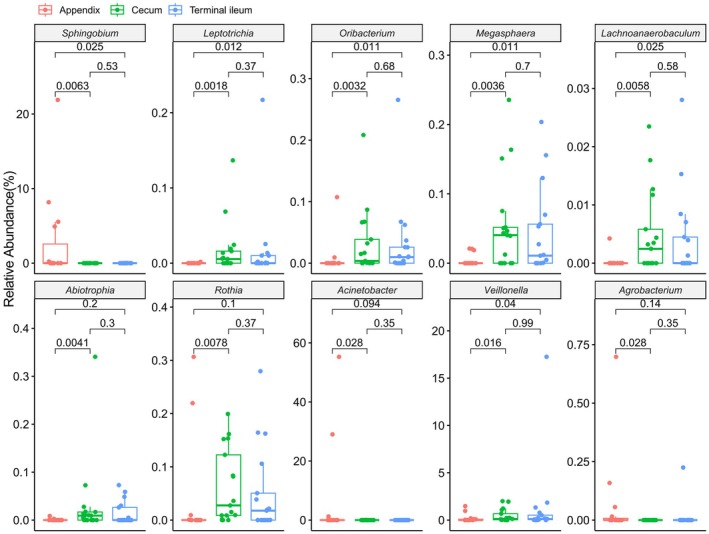
Genera variations in the abundance of microbiota in the appendix and its adjacent intestinal sites (Wilcoxon rank sum test, *P* < 0.05). In comparison with the cecum, the appendix exhibited a greater abundance of gut microbiota in several genera, including *Sphingobium* (*P* = 0.0063), *Leptotrichia* (*P* = 0.0018), *Oribacterium* (*P* = 0.0032), *Megasphaera* (*P* = 0.0036), *Lachnoanaerobaculum* (*P* = 0.0058), *Abiotrophia* (*P* = 0.0036), *Rothia* (*P* = 0.0078), *Acinetobacter* (*P* = 0.028), *Veillonella* (*P* = 0.016) and *Agrobacterium* (*P* = 0.028). Compared to the terminal ileum, the appendix demonstrated a higher prevalence of gut microbiota across several genera, including *Sphingobium* (*P* = 0.025), *Leptotrichia* (*P* = 0.012), *Oribacterium* (*P* = 0.011), *Megasphaera* (*P* = 0.011), *Lachnoanaerobaculum* (*P* = 0.025) and *Veillonella* (*P* = 0.04). Notably, no significant differences were observed for *Abiotrophia* (*P* = 0.2), *Rothia* (*P* = 0.1), *Acinetobacter* (*P* = 0.094) and *Agrobacterium* (*P* = 0.14). There was no statistically significant difference in microbial genera abundance between the cecum and the terminal ileum (*P* > 0.05).

**Fig. 5:**
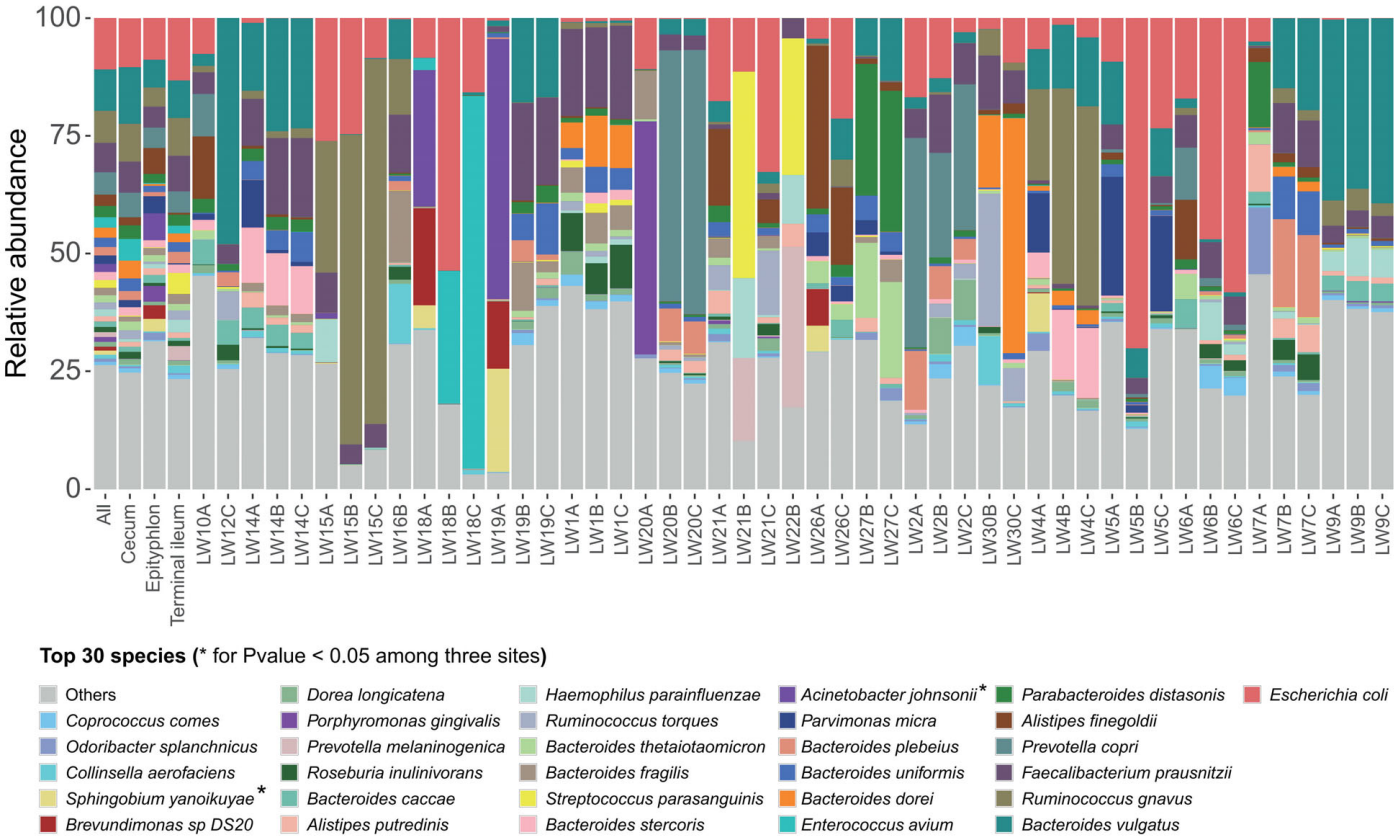
The top 30 species across the appendix, the cecum and the terminal ileum. *S. yanoikuyae* and *A. johnsonii* showed statistical significance (Kruskal–Wallis test, *P* < 0.05).

**Fig. 6:**
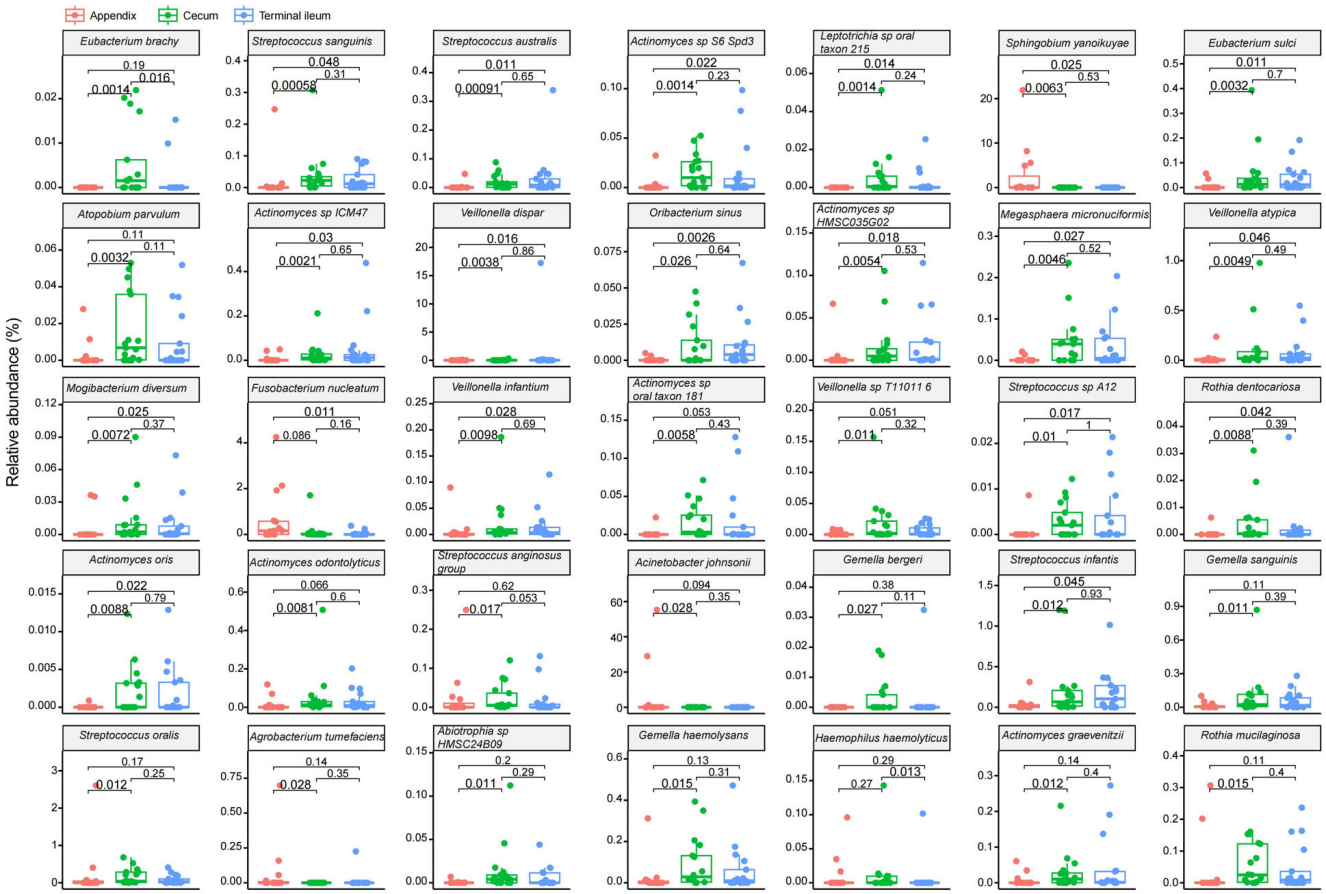
Species variations in the relative abundance of microbiota in the appendix and its adjacent intestinal sites (wilcoxon rank sum test, *P* < 0.05). At the species level, in contrast to the other sites, the appendix exhibited higher abundances of microbiota including *Sphingobium*, *Leptotrichia* and *Oribacterium* (*P* < 0.05), as well as a significant increase in species‐level abundance of oral bacteria, including *S. sanguinis* (*P* = 0.00058 and *P* = 0.048), *S. australis* (*P* = 0.00091 and *P* = 0.011), *Streptococcus* sp. A12 (*P* = 0.01 and *P* = 0.017), *Leptotrichia* sp. oral taxon 215 (*P* = 0.0014 and *P* = 0.014), *V. dispar* (*P* = 0.0038 and *P* = 0.016), *V. infantium* (*P* = 0.0098 and *P* = 0.011) and *O. sinus* (*P* = 0.026 and *P* = 0.0026), respectively. However, apart from *E. brachy* (*P* = 0.016) and *H. haemolyticus* (*P* = 0.013), no significant differences were found between the cecum and terminal ileum.

We conducted a comprehensive analysis of the top 30 genera (Kruskal–Wallis test) (Table S5) and species (Kruskal–Wallis test) (Table S6) across three sites. Among the genera, *Veillonella*, *Sphingobium* and *Acinetobacter* exhibited statistically significant differences (*P* < 0.05, Kruskal–Wallis test) (Fig. 3). Notably, there were significant variations in the abundance of gut microbiota genera between the appendix and its adjacent sites (*P* < 0.05, Wilcoxon rank sum test). Specifically, the appendix demonstrated higher genus‐level abundances for *Sphingobium* (*P* = 0.0063 and *P* = 0.025), *Veillonella* (*P* = 0.016 and *P* = 0.04) and *Acinetobacter* (*P* = 0.029 and *P* = 0.094) compared to the cecum and terminal ileum, respectively. However, no statistically significant differences were observed in microbial genera abundance between the cecum and the terminal ileum (*P* > 0.05) (Fig. 4). Furthermore, the top 30 species were analyzed, with *Sphingobium yanoikuyae* and *Acinetobacter johnsonii* showing statistical significance (*P* < 0.05) (Fig. 5). Compared to the other sites, the appendix exhibited higher abundances of microbiota including *Streptococcus sanguinis* (*P* = 0.00058 and *P* = 0.048), *Streptococcus australis* (*P* = 0.00091 and *P* = 0.011), *Streptococcus* sp. A12 (*P* = 0.01 and *P* = 0.017), *Leptotrichia* sp. oral taxon 215 (*P* = 0.0014 and *P* = 0.014), *Veillonella dispar* (*P* = 0.0038 and *P* = 0.016), *Veillonella infantium* (*P* = 0.0098 and *P* = 0.011) and *Oribacterium sinus* (*P* = 0.026 and *P* = 0.0026), separately. Apart from *Eubacterium brachy* (*P* = 0.016) and *Haemophilus haemolyticus* (*P* = 0.013), there were no significant differences between the cecum and terminal ileum (Fig. 6).

### Characteristics of the pathways in the appendix and its adjacent intestinal sites

**Fig. 7:**
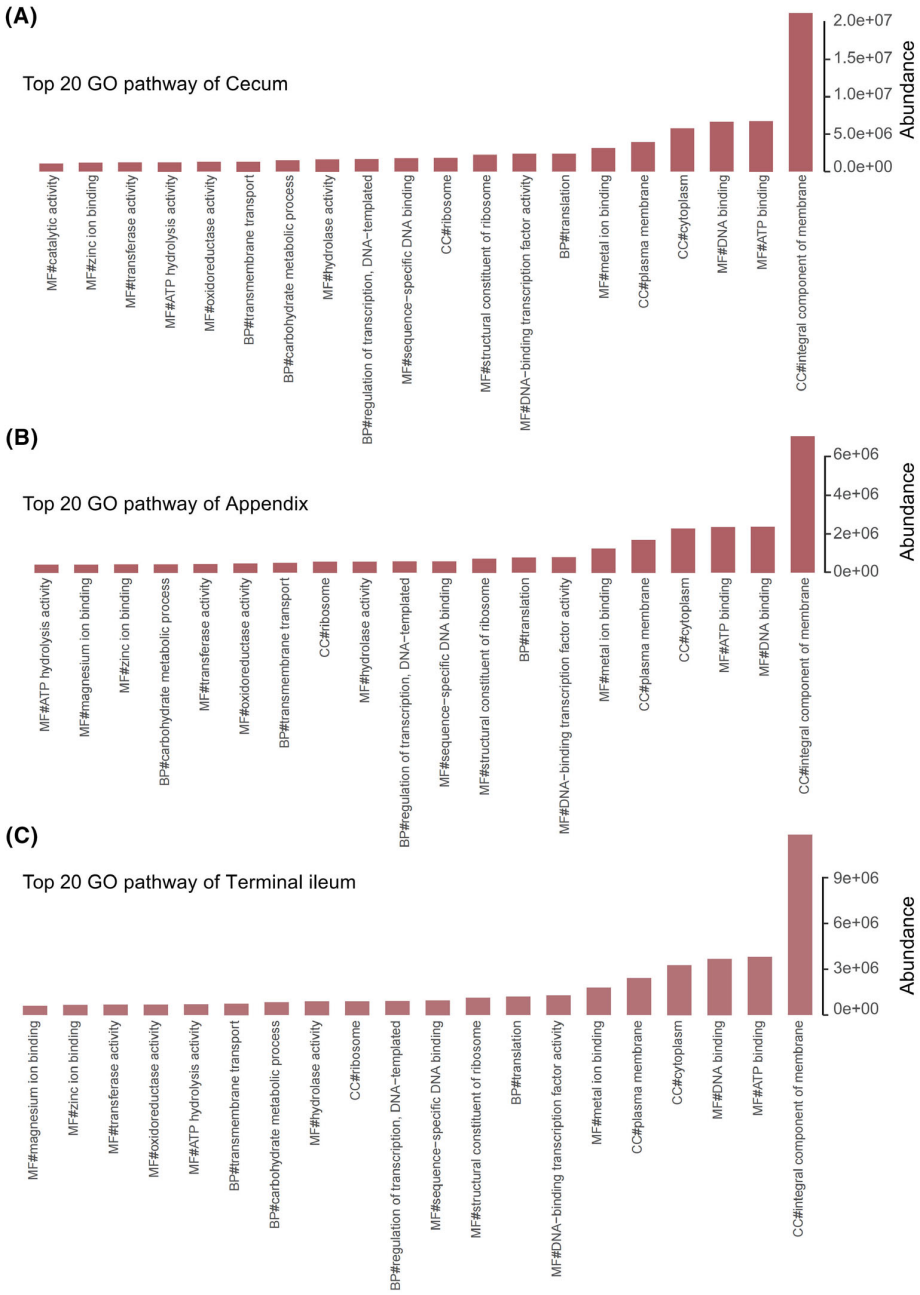
The top 20 GO pathways in the appendix, the cecum and the terminal ileum. (A) The top 20 GO pathways in the appendix. (B) The top 20 Gene GO pathways in the cecum. (C) The top 20 GO pathways in the terminal ileum).

**Fig. 8:**
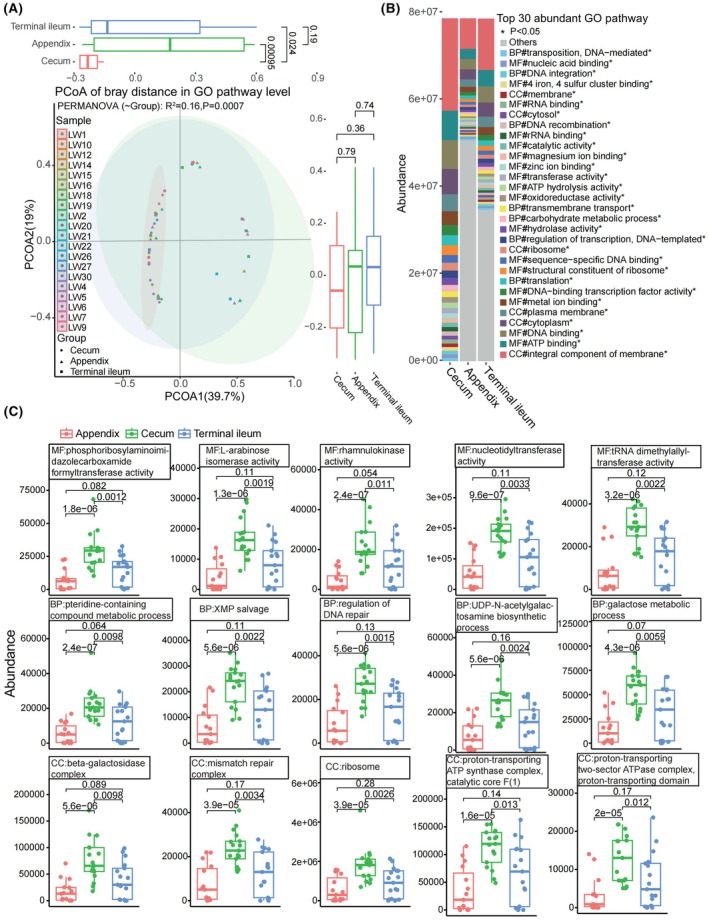
GO pathways and the β‐diversity in the appendix, cecum and terminal ileum. (A) Principal coordinate analysis of the Bray distance at the GO pathway revealed significant differences in microbiota composition among the three groups (PERMANOVA, *r* ^2^ = 0.16, *P* = 0.0007). (B) The top 30 GO pathways exhibited statistically significant differences in GO pathways (Kruskal–Wallis test, *P* < 0.05). (C) Significant differences in GO pathways among the three sites (Wilcoxon rank sum test, *P* < 0.05). The abundance of GO pathways was highest in the cecum, followed by the terminal ileum, and lowest in the appendix. Statistical analysis revealed significant differences between the appendix and cecum (*P* < 0.00001) and between the cecum and the terminal ileum (*P* < 0.05). However, no significant difference was observed between the appendix and the terminal ileum (*P* > 0.05).

We have compiled a list of the top 20 significant Gene Ontology (GO) (http://geneontology.org) pathways in the appendix, cecum and terminal ileum (Kruskal–Wallis test) (Fig. 7 and Table S7). Additionally, we analyzed the top 30 GO pathways that exhibited statistically significant differences (*P* < 0.05, Kruskal–Wallis test) among the three sites (Fig. 8B). Notably, the abundance of GO pathways was highest in the cecum, followed by the terminal ileum, and lowest in the appendix. Statistical analysis revealed significant differences between the appendix and cecum (*P* < 0.00001, Wilcoxon rank sum test) and between the cecum and the terminal ileum (*P* < 0.05). However, no significant difference was observed between the appendix and the terminal ileum (*P* > 0.05). For example, the top 5 significant differences of molecular function (MF) were: phosphoribosylaminoimi dazolecarboxamide formyltransferase activity, l‐arabinose isomerase activity, rhamnulokinase activity, nucleotidyltransferase activity and tRNA dimethylallyltransferase activity. The top 5 significant differences of biological process (BP) included: pteridine‐containing compound metabolic process, XMP salvage and regulation of DNA repair, UDP‐*N*‐acetylgalactosamine biosynthetic process and galactose metabolic process. The top 5 significant differences of cellular component (CC) consisted of the beta‐galactosidase complex, mismatch repair complex, ribosome, proton‐transporting ATP synthase complex, catalytic core F (1) and proton‐transporting two‐sector ATPase complex and proton‐transporting domain (Fig. 8C).

**Fig. 9:**
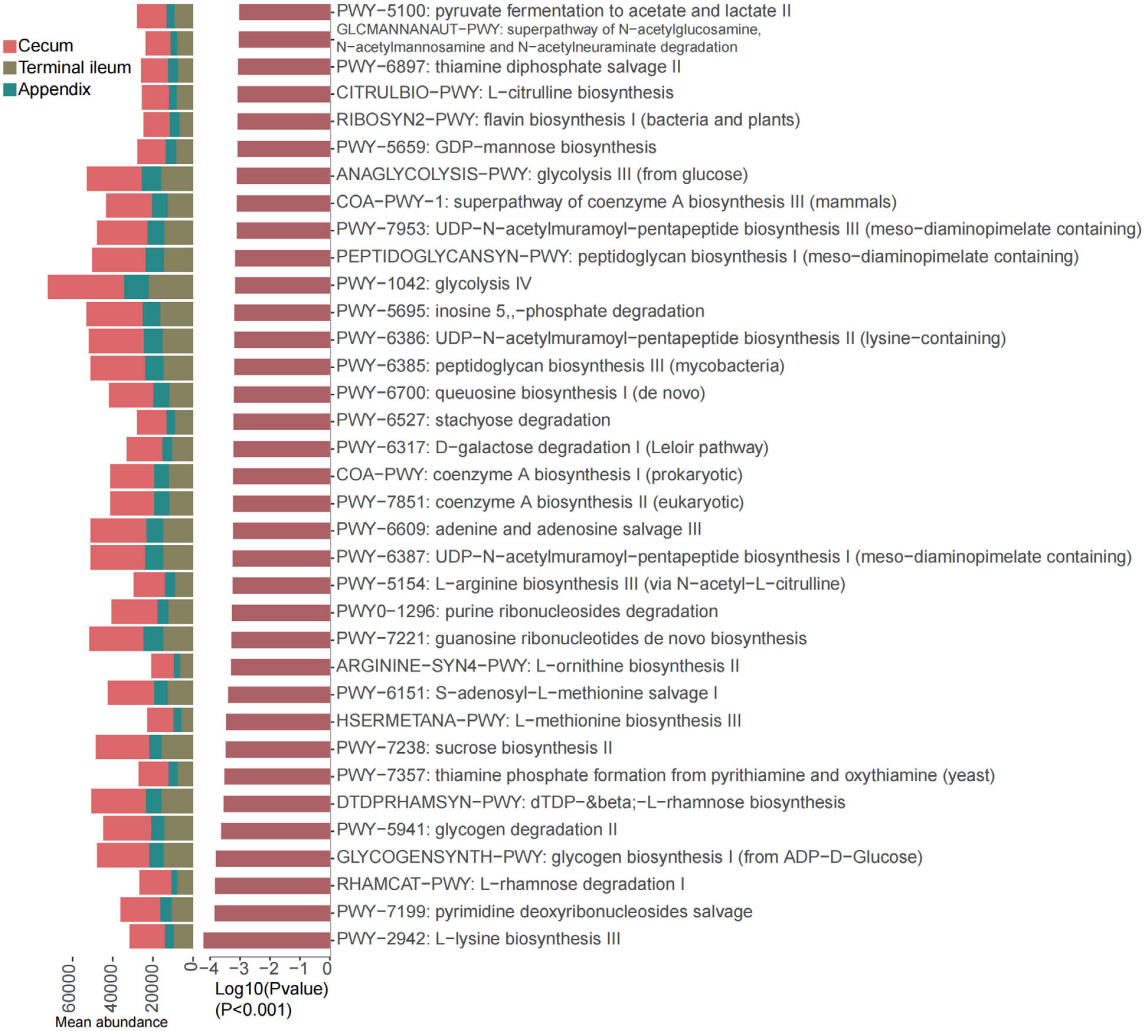
HUMAnN profiling revealed notable disparities among the appendix, cecum and terminal ileum across numerous pathways (Kruskal–Wallis test, *P* < 0.001). The cecal functions exhibited higher mean abundance compared to the other two sites (*P* < 0.001).

On the other hand, the profiling annotation from the UniRef database revealed notable pathways among the three sites (*P* < 0.001, Kruskal–Wallis test). Similar to the GO pathway profiling, the cecum exhibited the highest levels, followed by the terminal ileum and the appendix. The pathways with significant differences are listed (Fig. 9 and Table S8). The pathways involved in glucose metabolism included glycolysis IV, glycolysis III (from glucose), glycogen biosynthesis I, glycogen degradation II and d‐galactose degradation I (Leloir pathway). The nucleotide metabolism pathways were increased, such as UDP‐*N*‐acetylmuramoyl‐pentapeptide biosynthesis I, II and III. Additionally, the pathways of amino acid metabolism were also abundant, involving l‐methionine biosynthesis III, l‐ornithine biosynthesis II and l‐arginine biosynthesis III (via *N*‐acetyl‐l‐citrulline).

### Diversity analysis

The β‐diversity was calculated using the Bray–Curtis distance based on the taxonomic (Fig. S1) and GO pathway profiles (Fig. 8A). Principal coordinate analysis of the Bray distance at the GO pathway level revealed significant differences in microbiota composition among the three groups (*r* ^2^ = 0.16, *P* = 0.0007, PERMANOVA).

**Fig. 10:**
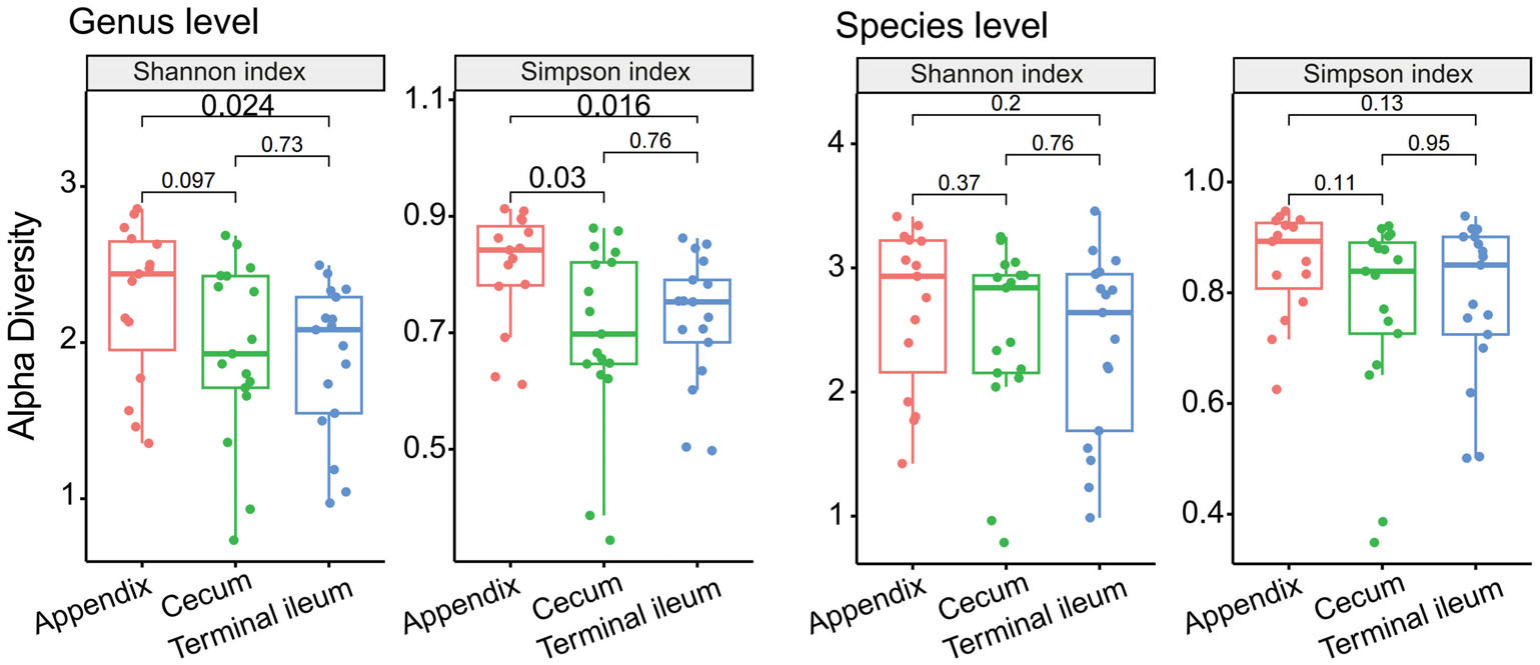
The α‐diversity of species and genus levels was assessed using the Shannon and Simpson indices (Wilcoxon rank sum test and Student's *t* test, *P* < 0.05). At the genus level, the *P* value in the Shannon and Simpson indices between the appendix and the terminal ileum was 0.024 and 0.016, respectively. The *P* value in these indices between the appendix and the cecum was 0.097 and 0.03, respectively. No variation in α‐diversity was observed between the cecum and the terminal ileum (*P >* 0.05). At the species level, α‐diversity did not exhibit significant differences among the appendix, cecum and terminal ileum (*P* > 0.05).

The α‐diversity of species and genus levels was assessed using the Shannon and Simpson indices (Table S9, Wilcoxon rank sum test and Student's *t* test). At the genus level, the *P* value in the Shannon and Simpson indices between the appendix and the terminal ileum was 0.024 and 0.016, respectively. The *P* value in these indices between the appendix and the cecum was 0.097 and 0.03, respectively. No variation in α‐diversity was observed between the cecum and the terminal ileum. At the species level, α‐diversity did not exhibit significant differences among the appendix, cecum and terminal ileum (Fig. 10).

### The relationship between species and functional differences

**Fig. 11:**
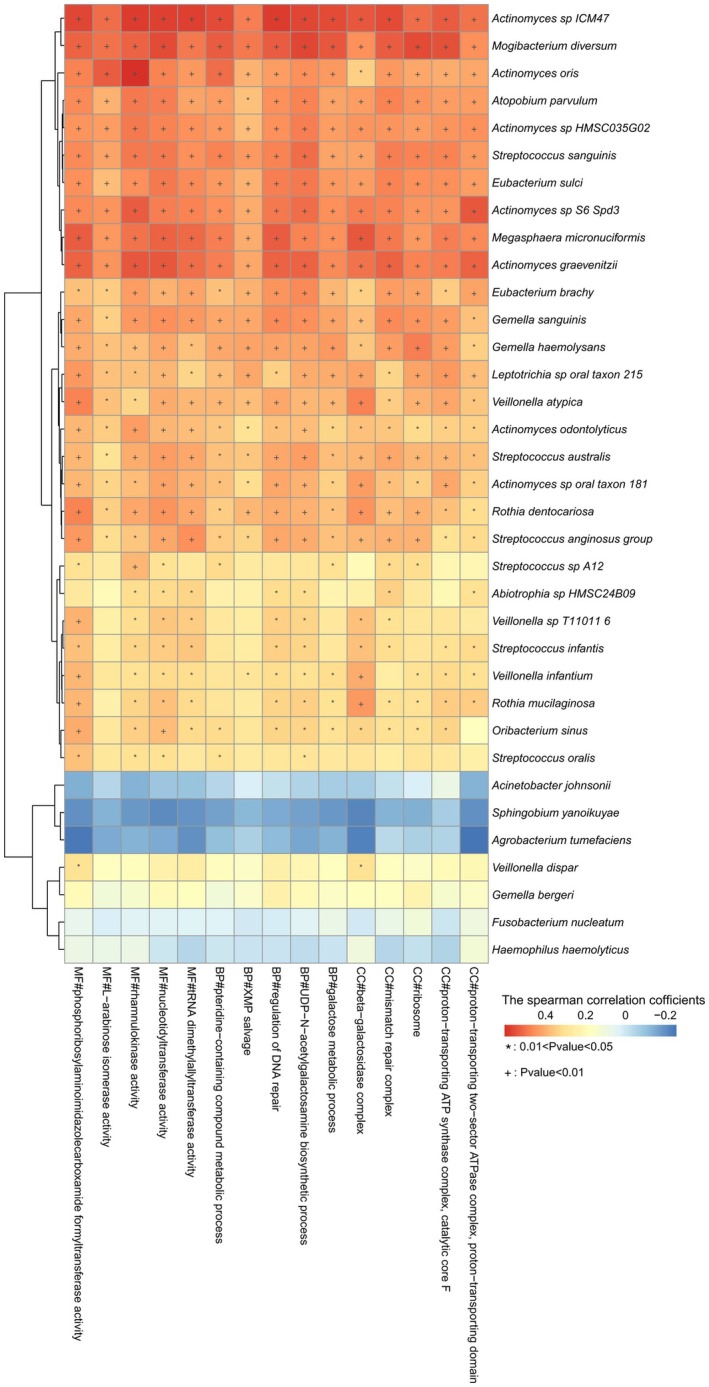
Correlation analysis between these species and the GO of significant differences. The species with a significantly higher mean relative abundance in the appendix included *A. johnsonii*, *S. yanoikuyae* and *A. tumefaciens*, showing negative correlations with top 5 abundant MF, CC and BP (Pearson correlation coefficient, *r* < 0, *P* > 0.05). The species *M. diversum*, *S. sanguinis*, *M. micronuciformis* and *A. graevenitzii*, etc., which were signficantly decreased in the appendix, showed significant positive correlations with the top 5 abundant MF, CC and BP (Pearson correlation coefficient, *r* < 0, *P* < 0.05).

To illustrate the correlation between different species and GO in more detail, we conducted correlation analysis between the species and the GO of significance (Fig. 11 and Table S10, Spearman correlation test). Certain species, including *A. johnsonii*, *S. yanoikuyae* and *A. tumefaciens*, had significantly higher mean relative abundance in the appendix than in the terminal ileum and cecum. These species were negatively correlated with the top 5 most abundant MF, CC and BP (Pearson correlation coefficient, *r* < 0, *P* > 0.05). The species *Mogibacterium diversum*, *S. sanguinis*, *Megasphaera micronuciformis* and *Actinomyces graevenitzii*, etc., which were signficantly decreased in the appendix, showed significant positive correlations with top 5 abundant MF, CC and BP categories (Pearson correlation coefficient, *r* > 0, *P* < 0.01).

## Discussion

Given the heterogeneous spatial distribution of gut microorganisms, studies adopting different sampling methods may yield varying results [37, 38, 39]. Most investigations have relied on stool samples [40], whereas appendiceal samples were primarily obtained from surgical specimens [21, 41, 42], including autopsy‐derived samples [43]. The present study first reports the heterogeneity in microbiota by sampling through the lumen of the inflamed appendix and its adjacent sites.

To further evaluate the ERAT sampling technique in the study of appendiceal microbiota, we compared it with the previous study by Guinane *et al*. [22] that utilized traditional appendectomy sampling (Table 1). In this regard, it was found that, at the phylum level, the top five phyla of the composition were the same, although the ranking of microbial abundance was slightly different (e.g. Bacteroidetes and Proteobacteria). Interestingly, both sampling methods identified oral bacteria. The study by Guinane *et al*. [22] was the first to comprehensively describe the bacterial composition of the human appendix using 16S rRNA sequencing, laying the groundwork for studying the microbiota's role in appendicitis. However, it was limited to 16S rRNA analysis, which provides only partial microbial information and lacks resolution at the species level. By contrast, the present study used metagenomic sequencing to generate full genomic profiles and enable more precise, species‐level analysis of the appendiceal microbiota. For example, the oral pathogens *Gemella*, *Parvimonas* and *Fusobacterium* were identified from the appendectomy specimens in Guinane's study, whereas species were also detected in our study as follows: *S. sanguinis*, *S. australis*, *Streptococcus* sp. A12, *Leptotrichia* sp. oral taxon 215, *V. dispar*, *V. infantium* and *O. sinus*, etc. Indeed, it has reported that the presence of oral bacteria in the intestinal microbiota may be associated with acute pediatric appendicitis, and it has been hypothesized that the oral microbiome could potentially serve as a relevant reservoir for acute appendicitis [44]. *Streptococcus* has been suggested to be associated with the development of acute appendicitis [45]. Oral biofilm infections involving *Streptococcus* have been correlated with inflammation and systemic diseases [46]. Toxins, proteins, nucleic acids and vesicles that are secreted within the biofilm may potentially contribute to the pathogenicity of oral *Streptococcus* [46], although their direct role remains to be fully elucidated. *Veillonella*, a bacterial genus found in both the oral cavity and gastrointestinal tract, has also been linked to gastrointestinal infections and associated with acute appendicitis [47, 48, 49]. Periodontal pathogens, such as *Fusobacterium nucleatum*, have been associated with an increased risk of Crohn's disease progression and exacerbation of colitis [50, 51]. *Actinomycetes* are involved in periodontitis and abdominal actinomycosis, with infections often associated with cold abscesses in inflammatory bowel disease (IBD) patients [52]. *Leptotrichia* species have been linked to conditions such as pneumonia, mucositis and sepsis [53]. Furthermore, atherosclerotic disease [54], rheumatoid arthritis [55], liver diseases [56], type 1 diabetes mellitus [57] colorectal cancer [58] and other disease are influenced by oral microorganisms. It has been proposed that the hematogenous route and the enteral route are potential pathways for the ectopic gut colonization by oral bacteria [59]. Similarly, in the present study, amounts of ‘beneficial bacteria’ or indicators of gut health were also detected similarly to those in the study by Guinane *et al*. [22]. For example, despite of the context of appendicitis, our findings indicated that there were short‐chain fatty acid (SCFA) producers such as *Bifidobacterium*, *Blautia*, *Roseburia* and *Facealibacterium* in the appendix. SCFAs are known play an important role in regulating both pro‐ and anti‐inflammatory processes. Studies show that SCFAs effectively inhibit intestinal commensals such as *E*scher*ichia coli* Nissle and enteric pathogens [60]. In IBD patients, reduced SCFA levels are linked to increased intestinal permeability and inflammation. Supplementing SCFAs or using probiotics that produce SCFAs may offer therapeutic potential for IBD [61]. SCFAs influence inflammatory responses through interactions with various signaling pathways and receptors, including histone deacetylases, G‐protein‐coupled receptor 109A, nuclear factor‐kappa B, free fatty acid receptors 2 and 3, and the mitigen‐activated protein kinase pathway. However, the exact mechanisms by which SCFAs modulate inflammation remain under investigation [62]. Especially, limited research has been conducted on the microbiota and metabolites in the inflamed appendix, let alone on the pathological and physiological mechanisms linking the microbiota to SCFAs. In our animal experiments, the positive effects of SCFAs on acute appendicitis were found (H. Ma, M. Wang, Q. Wang and X. Huang, unpublished data). Apart from sampling method differences, discrepancies in these two studies also stem from sequencing methodologies and the continuous updating of databases, which enhance the depth of microbiota detection. Other factors such as diet, antibiotic administration, and bowel preparation, that should be taken into the consideration [63, 64]. For example, a 24‐h fasting period preceded ERAT sampling, and none of the participants received antibiotics prior to sampling in this study. Conversely, in the study by Guinane *et al*. [22], antibiotics were administered preoperatively, potentially influencing microbiota composition. Furthermore, our study found no significant differences in factors such as age and Alvarado score, thereby reducing potential sample bias. Overall, our study detected much more microbial composition than the study by Guinane *et al*. [22], and may more accurately represent the true composition of the appendix microbiota. However, it should be noted that the sample sizes in both studies were inadequate. Future research should aim to validate these findings with larger sample sizes. Of course, comparisons with the study by Guinane *et al*. [22] are inter‐study, as a result of differences in sample collection, lab environments, instrument models, reagent batches and other factors, which may introduce batch effects.

**Table 1:** Comparison with the study by Guinane *et al*. [22].

	Guinane *et al*. [22]	Present study
Sample size (*n*)	7	20
Age range (years)	5–25	14–58
Antibiotics use or not	Yes	No
Sampling methods	Appendectomy	ERAT
Method of sequencing	16S rRNA (V4)	Shotgun metagenomic sequencing
Control of samples	Stool	Intestinal contents of the cecum and terminal ileum
Bowel cleansing or not	Not clear	Yes
Fasting or not	Yes	Yes
Characteristics of the microbial compositon		
Phylum level	Firmicutes Proteobacteria Bacteroidetes Actinobacteria Fusobacteria	Firmicutes Bacteroidetes Proteobacteria Actinobacteria Fusobacteria
Oral bacteria		
Genera level	*Gemella* *Parvimonas* *Fusobacterium*	*Parvimonas* *Fusobacterium*
Species level	–	*Streptococcus sanguinis* *Streptococcus australis*, *Streptococcus* sp. A12, *Leptotrichia* sp. oral taxon 215, *Veillonella dispar*, *Veillonella infantium* and *Oribacterium sinus*, etc.
Beneficial bacteria or indicators of gut health		
Genera level	*Bacteroides* *Lactobacillus* *Bifidobacterium*	*Bacteroides*, *Bifidobacterium*, *Blautia*, *Roseburia*, *Facealibacterium*, etc.

In the infectious diseases, substantial alterations in host metabolism, including hyperglycemia, perturbed amino acid metabolism and dysregulated lipid metabolism, have been correlated with marked changes in the levels and activity of cellular metabolites. Dysregulated lipid metabolism, in particular, has been identified as a potential factor that may contribute to immune dysfunction [65]. HUMAnN profiling revealed that the material and energy metabolism constituted an important part of metabolic pathways. Bacteria involved in glucose metabolism such as *Bifidobacterium*, *Faecalibacterium prausnitzii*, *Prevotella*, *Dorea*, *Bacteroides* and *Streptococcus* were abundant in the three sites. Taking *Bacteroides fragilis* as an example, it was reported to generate distinct capsular polysaccharide A, which possesses the dual capability to facilitate both pro‐inflammatory and anti‐inflammatory processes [66]. A recent study using a mouse model of cecal ligation and puncture has shown that gut commensal metabolite rhamnose is one of the key reactive bioactive metabolites improving the host's defense against sepsis [67] and we also found that the pathways for both the synthesis and degradation of rhamnose were significantly abundant in cases of acute appendicitis. Amino acid metabolism pathways were notably abundant at the three sites. It was also inseparable from the role of intestinal bacterium. For example, *Prevotella copri* and *Bacteroides vulgatus* have been identified as significant producers of branched‐chain amino acids, which are associated with the regulation of protein synthesis and glucose metabolism [68]. Additionally, an elevated abundance has been observed in the nucleotide metabolism pathway, which is linked to the generation of purine and pyrimidine molecules essential for DNA replication, RNA synthesis and cellular bioenergetics. Although these associations have been documented, the precise mechanisms by which these microbial metabolites influence host physiology remain areas of active investigation.

Interestingly, the appendix exhibited a higher abundance compared to the terminal ileum and cecum in terms of species and genera composition, whereas the cecum demonstrated a greater abundance in HUMAnN profiling and GO pathway representation than that in the terminal ileum and appendix. To better reveal the relationship between significant changes between GO pathways and taxonomic profiles, we have analyzed the correlation between significantly changed species and the top 5 abundant MF, CC and BP. Species that exhibited a significantly higher mean relative abundance in the appendix compared to the terminal ileum and cecum, such as *A. johnsonii*, *S. yanoikuyae* and *A. tumefaciens*, showed negative correlations with the top 5 abundant MF, CC and BP. The species *M. diversum*, *S. sanguinis*, *M. micronuciformis* and *A. graevenitzii*, etc., which were signficantly decreased in the appendix, showed significant positive correlations with top 5 abundant MF, CC and BP. These results suggested that the functional diversity of microorganisms abundant in the appendix appears to be lower compared to species with a higher mean relative abundance in the terminal ileum and cecum. The potential factors contributing to this phenomenon are outlined below: first, the intestinal environment, including pH levels, oxygen concentration and nutrient availability, may be associated with microbial growth and function [69]. Second, within microbial communities, interactions such as competition, symbiosis and antagonism may be related to microbial functionality. For example, two microbiotas that differ in composition (species and/or genes) may exhibit similar behaviors at higher functional levels, resulting in comparable protein and metabolite profiles [70]. Third, this phenomenon may be related to functional redundancy, that is, certain microbial functions can be performed by multiple species, which may help maintain overall functional stability even when the abundance of specific species fluctuates [71, 72]. Moreover, prediction uncertainty may also play a role. Although HUMAnN 3.0 is utilized to efficiently and accurately assess the abundance of functions, and the UniRef database is continuously updated, some microorganisms may possess uncharacterized functions, or certain functional genes may exist across multiple taxonomic units [35].

To our knowledge, the appendix is situated at the junction between the ileum and cecum. Its relative immunity to fecal flow, as a result of the protective function of the appendiceal valve, suggests its potential role as a microbial reservoir. Bowel preparation has been found to impact the abundance and diversity of gut microbiota [40]. Research has shown that the genera *Bacteroides* and *Veillonella* were more common in the bowel preparation group [73]. Additionally, it is important to consider that appendectomy status can hinder the reconstitution of certain bacterial genera after bowel preparation [74]. However, previous research has suggested that the appendix may protect important gut bacteria during gastrointestinal issues such as infections and diarrhea. In the present study, although participants diagnosed with appendicitis underwent bowel preparation before sampling, which likely caused a temporary imbalance in intestinal flora [75], the inflamed appendix unexpectedly exhibited significantly higher microbial abundance compared to the terminal ileum and cecum at both the species and genus level, and almost no significant differences were observed between the cecum and terminal ileum. Thus, all of these findings further substantiated the hypothesis that the appendix functions as a microbial ‘safe house’. However, it is uncertain whether the inflamed appendix still functions as a ‘safe house’. *Fusobacterium*, *Porphyromonas* and *Parvimonas* were abundant in the inflamed appendix in our research, whereas they have been considered as a potential contributor to IBD, possibly becaue of its ability to form biofilms both within and outside the appendix [76, 77]. This may also indicate that, during appendicitis, the proliferation of pathogenic bacteria, particularly oral bacteria such as *S. sanguinis*, *S. australis* and *O. sinus*, which colonize the appendix, may be associated with impairment of the function of the appendix as a ‘safe house’. This impairment, in turn, may influence the occurrence and progression of related diseases.

Certainly, our research does have some limitations. Because of the requirement that volunteers should not have used antibiotics before sampling, only 20 volunteers participated in our study, and quality filtering reduced the samples to 49 involving the three sites. The small sample size may limit the study of microbial composition, which is the biggest limitation of the present study. We will expand the sample size in future studies to make the research more convincing. Second, this appendiceal sampling method in our study required volunteers to undergo bowel preparation beforehand to facilitate endoscope insertion. However, bowel preparation may alter the microbiota composition and could potentially introduce bias into the results. To account for this, we also collected microbiota samples from adjacent sites for comparison. We found that the microbiota in the cecum and terminal ileum were more similar to each other, whereas the appendix lumen showed distinct differences. This suggests that bowel preparation has a smaller effect on the appendix microbiota compared to the intestinal microbiota. Third, sample contamination may occur during the sampling process. Therefore, during sampling, we improved the disposable spray catheter by inserting it into the endoscope biopsy channel to perform targeted sampling of the terminal ileum and cecum, and we used a disposable visual endoscope to sample the microbiota within the appendix lumen. These methods minimized sample contamination that could result from directly aspirating intestinal and appendiceal contents through the biopsy channel. In addition, the supplementation of longitudinal study data to make the study more in‐depth will also be one of the key tasks in our follow‐up study.

Compared with the conventional sampling methods for appendectomy specimens, this novel approach may enable dynamic monitoring of microorganisms within the appendix lumen, thereby facilitating longitudinal studies of the appendix microbiota. Currently, the microbiological analysis of acute appendicitis in clinical practice is primarily conducted through microbial cultures obtained from the blood of patients with bacteremia or from purulent specimens of the appendix. The selection of antibiotics for treating appendicitis generally relies on broad‐spectrum agents that are effective against common pathogens. However, if a precise analysis of the appendix microbiota composition can be performed based on metagenomic detection results (e.g. identifying an increased abundance of oral‐derived pathogenic bacteria within the appendiceal lumen), targeted antibiotic therapy directed against these specific microorganisms could possibly be employed to effectively manage the condition. Therefore, through in‐depth investigation of the microbiota in the appendix cavity, clinicians might be able to formulate precise microbial treatment strategies based on changes in the microbial composition of the appendix lumen. Furthermore, the implementation of this sampling technique could potentially provide valuable references for diagnosing and treating other diseases associated with the regulation of the appendix microbiome, not only in appendicitis.

In conclusion, the present study initially employed an innovative sampling technique facilitated by ERAT, thereby enhancing the accuracy of microbiome profiling in the appendix niche of patients with acute appendicitis. Collectively, investigating the characteristics and functions of microbiota in appendicitis remains clinically valuable for guiding antibiotic use in both conservative management and surgical intervention of acute appendicitis. Specifically, the increased abundance of oral bacteria in the appendix is particularly significant for further elucidating the pathogenesis of acute appendicitis.

## Conflicts of interest

The authors declare that they have no conflicts of interest.

## Author contributions

HM and MW were responsible for investigations. HM, MW, GK, JF, PW and YF were responsible for methodology. HM, MW, WZ, WW, YX, GK, JF and PW were responsible for writing the original draft. WZ, WW and YX were responsible for formal analysis. YF QW and XH were responsible for reviewing and editing. QW was responsible for data curation and software. XH was responsible for conceptualization, project administration and supervision.

## Data Availability Statement

The original contributions presented in the study are publicly accessible at: https://urldefense.com/v3/__https://db.cngb.org/data_resources/project/CNP0007870/__;!!N11eV2iwtfs!uwryHLWX96T8sVOiIcgDc66PL2nBia_Fw-BlS4AihUGH8MxpH6eqABtjwe8WhfPYC_emv6qRD5FgBNPFKjxw$.

## Supporting information


**Fig. S1.** The β‐diversity was calculated using the Bray–Curtis distance based on the genus level. There was no significant difference among the appendix, cecum and terminal ileum (permutational multivariate analysis of variance test, *P* > 0.05).

**Table S1.** Information on patients' phenotype.
**Table S2.** Information on sequencing data.
**Table S3.** PERMANOVA of phenotype and taxon at the genus, species and pathway level.
**Table S4.** Relative abundance of all phyla.
**Table S5.** Relative abundance of all genera.
**Table S6.** Kruskal–Wallis rank sum test.
**Table S7.** Abundance of the GO pathway.
**Table S8.** Abundance of the UniRef pathway.
**Table S9.** Alpha diversity.
**Table S10.** Correlation between species and pathway.

## Data Availability

The data that support the findings of this study are openly available at the China National GeneBank Database (CNGBdb) with accession number: CNP0004326 (https://db.cngb.org/search/project/CNP0004326). The data have also been uploaded to NCBI SRA with accession number: PRJNA1282460, ID: 1282460.
