# Supplementary material for: Microbial profile of the appendix niche in acute appendicitis: a novel sampling approach
Source: FEBS Open Bio. 2025 Aug 22;15(12):2001–20. doi: 10.1002/2211-5463.70105 (PMC12667213; doi:10.1002/2211-5463.70105)

Supplementary Figure1. The β-diversity was calculated using the Bray-Curtis distance based on the genus level. It was no significant difference among the appendix, cecum and the terminal ileum. (Permutational multivariate analysis of variance test, P＞0.05)

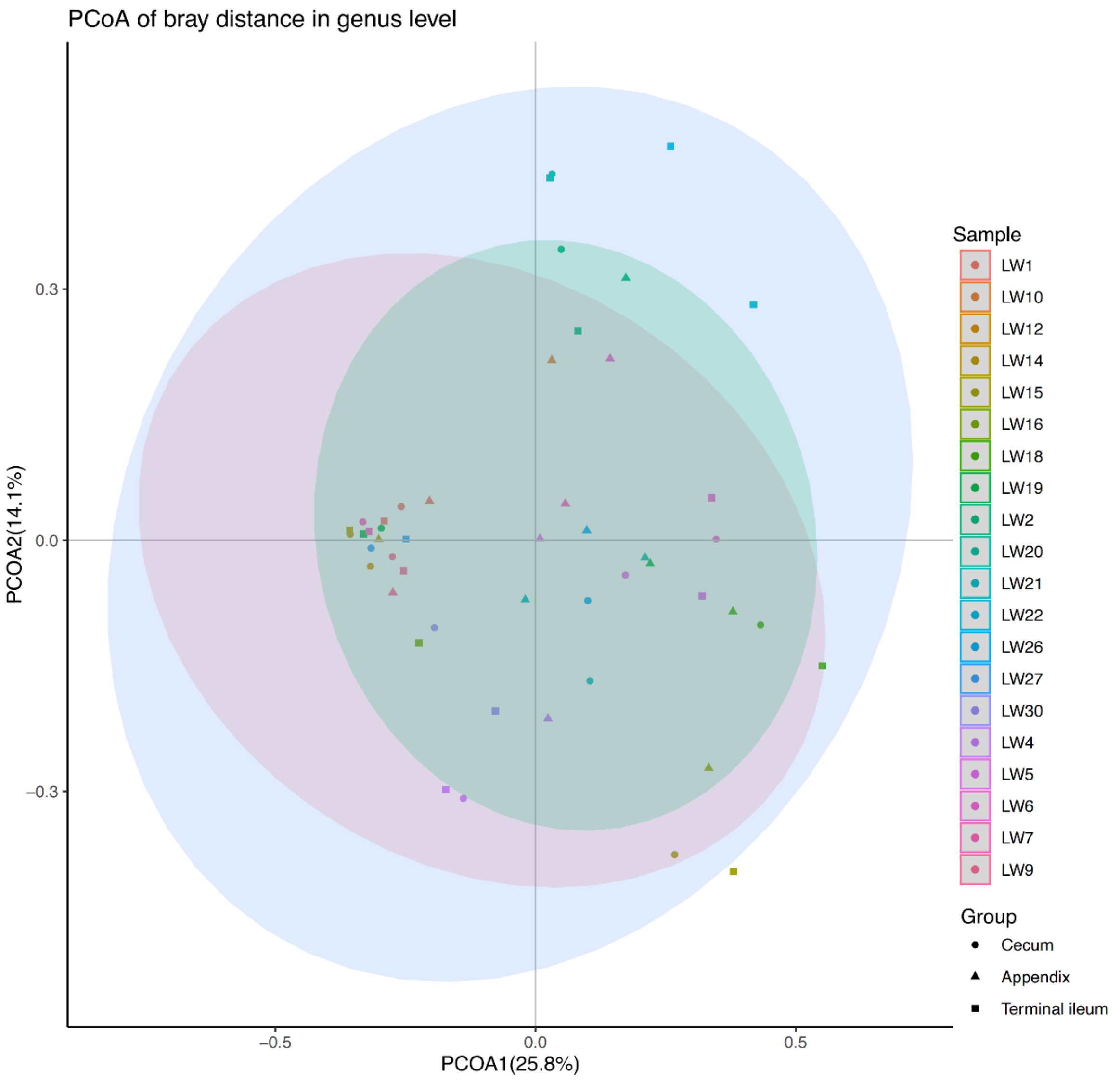

Supplement: Supplementary file 1 — Fig. S1. The β‐diversity was calculated using the Bray–Curtis distance based on the genus level. There was no significant difference among the appendix, cecum and terminal ileum (permutational multivariate analysis of variance test, P > 0.05). [file FEB4-15-2001-s002.pdf]
